# Hearing and Balance Exceed Initial Bone Mineral Density in Predicting Incident Fractures: A 25‐Year Prospective Observational Study in Menopausal Women With Osteoporosis

**DOI:** 10.1002/jbm4.10551

**Published:** 2021-09-30

**Authors:** Annika Dotevall, Emily Krantz, Marie‐Louise Barrenäs, Kerstin Landin‐Wilhelmsen

**Affiliations:** ^1^ Institute of Medicine, Sahlgrenska Academy University of Gothenburg Gothenburg Sweden; ^2^ Department of Medicine Sahlgrenska University Hospital/Östra Gothenburg Sweden; ^3^ Department of Respiratory Medicine and Allergology Sahlgrenska University Hospital Gothenburg Sweden; ^4^ Department of Physiology, Institute of Neuroscience and Physiology, Sahlgrenska Academy University of Gothenburg Gothenburg Sweden; ^5^ Department of Clinical Sciences, Division of Otorhinolaryngology Umeå University Umeå Sweden

**Keywords:** BALANCE, FRACTURES, HEARING, OSTEOPOROSIS, WOMEN

## Abstract

Hearing and balance deteriorate, and fracture incidence increases with age, especially in women. The aim of the present study was to investigate whether impaired hearing and body balance are stronger predictors of fractures than bone mass. Between 1995 and 1997, 80 women, aged 50 to 70 years, with primary osteoporosis, taking menopausal hormone therapy, mainly for menopausal symptoms, participated in a double‐blind, randomized, placebo‐controlled study of treatment with growth hormone versus placebo. All women received calcium 750 mg and vitamin D 400 U daily. They were then examined yearly until 2007 and followed up by registers until 2020. Hearing was assessed by audiometry. Body balance and fine motor function were tested according to the Bruininks‐Oseretsky test. Bone properties were measured with DXA. Data on fractures were derived from the Gothenburg Hospital register. Over the 25‐year follow‐up, 50 women (63%) sustained 104 fractures, most often related to accidental falls. Thoracic and lumbar spine fractures were most common (36%). Other fractures occurred in the pelvis (14%), humerus (14%), hip (11%), and wrist (10%). Hearing impairment at baseline, measured as pure tone average‐high (*p* = 0.007), pure tone average‐mid (*p* = 0.003), and speech‐recognition score (*p* = 0.025), was associated with a subsequent first fracture, as were worse body balance (*p* = 0.004), upper limb coordination (*p* = 0.044), and higher running‐speed agility (*p* = 0.012). After adjustment for age and BMD, pure tone average‐high (*p* = 0.036), pure tone average‐mid (*p* = 0.028), and body balance (*p* = 0.039) were still significantly associated with incident fractures. Bone mineral content, BMD, and treatment at baseline were not associated with subsequent fracture. In conclusion, hearing and body balance at baseline exceeded initial BMD in predicting incident fractures in osteoporotic women regardless of treatment during 25‐year follow‐up. © 2021 The Authors. *JBMR Plus* published by Wiley Periodicals LLC on behalf of American Society for Bone and Mineral Research.

## Introduction

The risk of fractures increases with age, especially in women after menopause. A Canadian study reported a 24% 10‐year absolute risk of fragility fractures in 75‐ to 84‐year‐old women.^(^ ^1^ ^)^ The most common fragility fractures are of the hip and radius. Both are related to falling and are associated with increased mortality in the short and long term.^(^ ^2^, ^3^, ^4^ ^)^ The risk of sustaining a fracture is further greatly increased by the presence of osteoporosis, defined as low BMD or low bone mineral content (BMC).^(^ ^5^ ^)^

Impaired balance influences the risk of falling and thereby the risk of fractures. In a previous study, we showed that impaired hearing predicted incident fractures in a general population sample of middle‐aged and elderly Swedish women (up to 98 years of age) followed up for 17 years. In contrast, bone‐regulating hormones, medication, and lifestyle factors did not predict incident fractures.^(^ ^6^ ^)^

The present study is a follow‐up of 80 women with osteoporosis who participated in a double‐blind, randomized, placebo‐controlled study of treatment with growth hormone (GH) or placebo between 1995 and 1997. At the end of the 3‐year intervention and at follow‐up after 5 years, BMC had increased significantly in a dose‐dependent manner in women treated with GH compared with placebo.^(^ ^7^ ^)^ However, at the 10‐year follow‐up, BMC had decreased to levels similar to pretreatment levels, and there was no difference in the fracture incidence between women in the GH and placebo groups.^(^ ^8^ ^)^ The aim of this study was to investigate if body balance and fine motor function, beside impaired hearing, at the start of the study period were stronger predictors of a subsequent first fracture and death than bone mass and treatment in these women with osteoporosis 25 years later.

## Materials and Methods

### Subjects

Eighty women aged 50 to 70 years, with primary osteoporosis, were recruited from the Endocrine Outpatient Clinic from 1994 to 1995, from consultants in the city, and by advertisements in the local newspaper. The trial design has been described in detail previously.^(^ ^7^ ^)^ Osteoporosis was defined according to the World Health Organization (WHO) as BMD lower than −2.5 SD of young women (*T*‐score) from the Lunar, General Electric Healtcare, Dual energy X‐ray Absorptiometry (DXA), USA Reference Population, measured at the lumbar spine.^(^ ^5^ ^)^ Exclusion criteria were diabetes mellitus, ischemic heart disease, heart failure, cancer, kidney disease, or other chronic diseases, including chronic disease of the skeleton, as well as treatment with corticosteroids or osteoclast inhibitors. Out of 451 women taking part in the initial screening for osteoporosis, 371 did not meet the inclusion criteria, mainly based on not having osteoporosis. There were 77 women with osteoporosis, according to the WHO criteria, who were included. Because of difficulties in recruiting a sufficient number of participants, another three women with a higher BMD (<−2 SD as *T*‐score), but with at least one previous osteoporotic fracture were included. All women were menopausal and at least 1 year had passed since their final menstruation. They had been treated with menopausal hormone therapy (MHT) for at least 9 months before inclusion in the study, the majority based on menopausal symptoms.

The women were randomized into a double‐blind, placebo‐controlled study with growth hormone (GH) in two doses, 1.0 U/day (n = 28) or 2.5 U/day (n = 27) versus similar volumes of placebo (n = 25) subcutaneously from 1995 until 1997. The double‐blind phase lasted 18 months. Thereafter, all women who received GH continued the injections for another 18 months, altogether 3 years. Based on ethical considerations, those receiving placebo injections stopped at 18 months. All participants received 750 mg of calcium and 400 U of vitamin D daily during the trial and follow‐up. The study was performed at the Center for Endocrinology and Metabolism at Sahlgrenska University Hospital, Gothenburg, Sweden.

The same investigator (K L‐W) examined all women yearly for 7 years after the GH‐treatment was stopped, altogether 10 years. There were no dropouts during this period except for six women who died.^(^ ^8^ ^)^ The present study reports incidence of the first fracture and mortality among the 80 women initially participating in the GH study up to 2020: a follow‐up of 25 years.

### Questionnaires and physical examination

At the start of the study in 1995, the participating women answered a questionnaire on past and present health status, previous fractures, medication, smoking habits, and physical activity. Medication was coded according to the Anatomical Therapeutic Chemical (ATC) classification system. Information about the use of bone‐specific treatment during follow‐up after the GH trial was obtained from medical records. Smoking habits were defined as current smokers or nonsmokers (i.e., never or former smokers).

Physical activity was coded on a four‐grade scale, with: (i) representing sedentary activity; (ii) moderate activity; (iii) regular, strenuous activity; and (iv) regular, very strenuous activity. The two latter categories were combined into one because there were very few women in category iv . The grading was based on a physiological analysis of exercise demands.^(^ ^9^ ^)^ Very few of the participating women were working full‐ or part‐time, so only physical activity during leisure time was analyzed.

Body weight was measured barefoot in light underwear to the nearest 0.1 kg. Body height was measured barefoot with a stadiometer to the nearest cm. BMI was calculated as body weight divided by height squared (kg/m^2^).

### Manual coordination

The Bruininks–Oseretsky test (BOT)^(^ ^10^ ^)^ subtests, upper limb coordination and manual dexterity were used. Upper limb coordination was assessed by (i) catching a tossed ball with both hands and thereafter preferred hand—5 trials each, (ii) catching a bounced ball with both hands and preferred hand—5 trials each, (iii) touching the nose with the index finger with closed eyes, (iv) by touching the thumb to the fingertips with closed eyes, and (v) by pivoting the thumb and index finger (90 seconds maximum for items iii–v). The maximum score was 21 points.

In the manual dexterity subtest, the subjects performed five timed actions: (i) the number of coins placed into a box with preferred hand in 15 seconds, (ii) time to place 12 pairs of coins in two boxes with both hands simultaneously, (iii) the number of shape cards sorted with preferred hand in 15 seconds, (iv) the number of beads strung with preferred hand in 15 seconds, and (v) the number of pegs placed on a pegboard with preferred hand in 15 seconds. The maximum outcome for each item was 8, 10, 10, 7, and 8 points, respectively, giving a maximum score of 43 points.

### Body balance

Body balance was measured using the BOT subtest running‐speed agility and a balance test using a modification of the BOT subtest balance. Running speed agility was recorded by a 25‐m shuttle run in a corridor assessed as time in seconds: the faster the run, the higher the point score. The balance score included measures of both static and dynamic balance. In all the tests, the subject was allowed to use her arms for balance (i.e., hands were not necessarily on the hips). Static balance was determined as the total score from six items: Romberg's test, the toe‐to‐heel Romberg's test, and standing on the preferred foot with the free (nonpreferred) leg flexed at the standing knee, with eyes open and closed for 30 seconds. The seconds before the subject put the nonpreferred foot down were recorded. Balance was also tested by using item 5 (the subject walks forward on a balance beam using a normal stride), and item 7 (the subject walks forward on a balance beam using a toe‐to‐heel gait) from the BOT protocol. The beam was made up of four parts and the subject scored 1 point if only the first part was passed and 4 points if all four parts were passed. If the subject was unable to reach the maximum time on the first trial of each item, two more trials were permitted, and the best result used. The rough score of the numbers of seconds was then exchanged for a point score (Supplementary Information S1).^(^ ^11^ ^)^ The maximum outcome for the static balance subtests was 29 points and for the dynamic balance tests—8 points, giving a total of 37 points: the better the result, the higher the score. All balance tests were performed by the same physician (M‐LB).

### Body composition analysis

BMD (g/cm^2^), BMC (kg), body fat, and lean body mass were measured with DXA (LUNAR DPX‐L; Lunar Radiation Inc), including total body, lumbar spine (anteroposterior [AP] L_2_–L_4_), femoral neck, and distal radius. LUNAR software was used for scanning (version 1.33) and analysis (version 1.33). In‐house precision errors on the scanner used (system 7156), as determined from duplicate examinations in 10 healthy subjects, were 1.46% for total body BMD, 0.81% for AP spine BMD, 1.25% for femoral neck BMD, and 1.66% for forearm BMD. The corresponding variation for total body BMC was 1.94%. The reference database used was the LUNAR USA Reference Population for the region examined. A quality assurance test with a phantom was performed every day of the study. The SD for repeated measures was 0.01 g/cm^2^ (1%) for L_2_–L_4_ and 0.015 g/cm^2^ (1.5%) for femoral neck during both short‐ and long‐term registrations.^(^ ^7^ ^)^

### Audiometry

All hearing tests were performed according to internationally accepted procedures (ISO 8253–1, 1989) by audiologists. The audiometers (Interacoustics AC‐30, Madsen OB‐822) were regularly calibrated in accordance with ISO 389 (1991), using TDH‐39 earphones with MX‐41/AR cushions. Two pure‐tone averages (PTAs) for both ears merged were used as summary statistics: PTA‐High (PTA at 3, 4, and 6 kHz) and PTA‐Mid (PTA at 0.5, 1, and 2 kHz).

Speech audiometry was assessed by performing the speech recognition score in noise, where the percentage of correct answers out of 50 monosyllabic phonemically balanced (PB) words is determined using a speech‐weighted noise and a signal‐to‐noise ratio of +4 dB HL. The test material consisted of six Swedish phonemically balanced word lists, taken from test material commonly used in Swedish speech audiometry practices.^(^ ^12^, ^13^ ^)^

### Fractures and mortality

The fractures were coded according to the *International Classification of Diseases‐10* (*ICD‐10*):^(^^14)^ fractures of the vertebrae including thoracic and lumbar spine M48.5, S22.0, and pelvis S32.0, ribs S22.3–4, humerus S42.2–4, forearm S52.0–4, S52.7, wrist S52.5–6, hand S62.0–8, hip S72.0–2, thigh S72.3–9, lower leg S82.1–4, ankle S82.5–6, S82.8, and foot S92.0–9. In addition, a lumbar x‐ray was performed in all subjects at the start of the study, then after 3 and 10 years. If the vertebral height declined >20% (according to the Genant classification) it was considered a new vertebral fracture.^(^ ^15^ ^)^ X‐ray‐verified fractures were thereafter retrieved until December 31, 2019 by searching the Gothenburg Hospital register. How and when each fracture occurred was asked at every visit. Only the first fracture that was considered osteoporotic was included in the analysis.

Date of death was retrieved from the Gothenburg Hospital register.

### Statistical methods

Conventional methods were used for the calculation of means and SDs. For comparison between groups, the Mantel–Haenszel χ^2^ test was used for ordered categorical variables and the Fisher's nonparametric permutation test was used for continuous variables. The Cox proportional hazard model was used to predict factors of importance for a future first fracture after the study's start. Subsequent fractures were not analyzed. The proportional hazard assumption was tested by including time‐dependent covariates in the Cox model. The Bonferroni correction method was performed to adjust for multiple testing. The results were censored at death. A *p* < 0.05 was considered significant.

### Ethical considerations

The University of Gothenburg Ethics Committee approved the study protocol (D‐number 386‐92 approved February 11, 1993; S543‐00 approved January 3, 2001; D‐number 2019‐05675 approved January 30, 2020). The study was performed in conformity with the Declaration of Helsinki.

## Results

Table 1 shows baseline variables for the participating women. There was no difference in mean age, prevalence of previous fractures, lifestyle factors, menopausal age, duration of MHT, smoking, or GH‐treatment between women who did and did not sustain a fracture during the follow‐up. There was a trend, albeit not significant, of higher BMI (24.3 vs 23.0 kg/m^2^) in the women who had sustained a fracture.

**Table 1:** Baseline Variables in 80 Women With Osteoporosis With and Without a Fracture During 25 Years of Follow‐Up

Variable	No fracture (n = 30)	Fracture (n = 50)	*P* Value
Age, y	60.3 (5.7)	61.4 (6.0)	0.43
Previous fracture, No. (%)	13 (43)	28 (56)	0.10
Body weight, kg	62.2 (7.2)	64.9 (7.6)	0.12
Height, m	1.64 (0.06)	1.64 (0.06)	0.63
BMI, kg/m^2^	23.0 (2.7)	24.3 (3.0)	0.058
Physical activity, sedentary, No. (%)	2 (7)	8 (16)	0.051
Menopausal age, y	49.2 (3.4)	48.1 (5.5)	0.38
Duration of estrogen treatment at baseline, y	5.4 (5.8)	5.6 (5.5)	0.87
GH treatment 1995–1997, No. (%)	18 (62)	36 (72)	0.50
Bone‐specific treatment after the GH trial, No. (%)	13 (43)	29 (58)	0.10
Smoking, No. (%)	6 (20)	14 (28)	0.37
Body fat, kg	21.3 (6.5)	24.2 (7.1)	0.08
Lean body mass, kg	38.5 (3.1)	39.0 (3.0)	0.50
BMC, kg	2.03 (0.23)	1.99 (0.25)	0.41
BMC femoral neck, kg	3.64 (0.65)	3.38 (0.78)	0.13
BMC L_2_–L_4_, kg	36.5 (5.7)	34.6 (6.1)	0.18
BMD, g/cm^2^	0.984 (0.059)	0.971 (0.072)	0.43
BMD femoral neck, g/cm^2^	0.785 (0.108)	0.748 (0.102)	0.13
BMD, L_2_–L_4_, g/cm^2^	0.886 (0.080)	0.860 (0.109)	0.26

Means (SD) are given for continuous variables. Categorical variables are presented as No. (%). For comparison between groups, the Mantel–Haenszel χ^2^ test was used for ordered categorical variables and the Fisher's nonparametric permutation test was used for continuous variables.

BMC = bone mineral content; BMD = bone mineral density; l = lumbar spine.

**Fig. 1:**
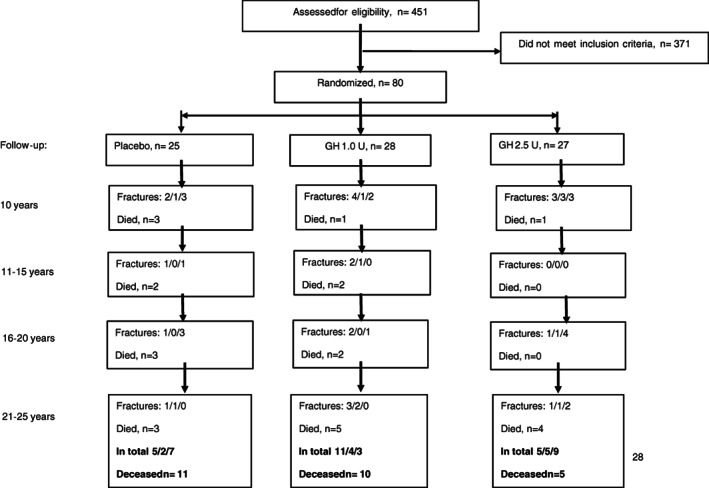
Flow chart depicting the initial 80 women who participated in a randomized, placebo‐controlled, double‐blind study with growth hormone (GH) subcutaneously daily for 3 years^(^ ^7^ ^)^ followed for 25 years. Number of women who suffered from a fracture (vertebral/hip/peripheral) and number of deaths are included.

All 80 women completed the 3‐year study with GH/placebo. A flow chart including number of women with fracture and type of fracture is given in Fig. 1. Twenty‐six women died during the 25‐year follow‐up, evenly distributed in the treatment groups. Of these, four women died within 1 year after a fracture (spine, spine + foot, upper arm, and lower leg). None of the deceased women had a femoral fracture during their last year of life. There was no difference in hearing or manual coordination at baseline between the women who died and those who did not during the follow‐up, but body balance was worse among those who died during the follow‐up (*p* < 0.001).

Fifty women (63%) sustained a fracture during the 25‐year follow‐up. Twenty‐seven women had one fracture, 11 had two, five had three, and seven women had more than three fractures; one woman sustained seven different fractures on separate occasions. Of the 104 fractures, thoracic and lumbar spine fractures were most common (36%). Other major fracture types were pelvic (14%), humerus (14%), hip (11%), and wrist (10%) fractures. Most fractures were caused by accidental falls.

There was no significant difference in body fat, lean body mass, BMC, or BMD among women with and without a subsequent fracture (Table 1).

Hearing was worse in women with a subsequent fracture (Table 2). Speech recognition score in noise was significantly poorer (*p* = 0.040), and there was a trend, although not significant, of higher PTA, indicating a more impaired hearing, in women who fractured during follow‐up. Body balance was significantly worse in women who fractured (*p* = 0.036; Table 2).

**Table 2:** Audiometry and Balance Variables at Baseline in 80 Women With Osteoporosis With and Without a Fracture During 25 Years of Follow‐Up

Variable	No fracture (n = 30)	Fracture (n = 50)	*p* Value
PTA‐high, dB	22.8 (12.1)	28.5 (16.0)	0.10
PTA‐mid, dB	11.8 (6.5)	15.9 (12.3)	0.11
Speech recognition score in noise, by 1%	76.5 (7.4)	68.4 (20.5)	0.040
Body balance, by score	17.1 (5.0)	13.8 (7.0)	0.036
Running‐speed agility, by score	11.3 (4.0)	12.6 (3.4)	0.16
Upper‐limb coordination, by score	14.2 (1.4)	13.5 (2.4)	0.18
Manual dexterity, by score	27.4 (3.9)	25.7 (4.3)	0.10

Means (SD) are given. For comparison between groups, the Fisher's nonparametric permutation test was used for continuous variables.

PTA‐high = pure tone average‐high at 3, 4, and 6 kHz; PTA‐mid = pure tone average‐mid at 0.5, 1, and 2 kHz.

Hazards ratios (HRs) for a fracture during the 25‐year follow‐up are given in Table 3. The unadjusted risk was significantly associated with hearing impairment, measured as PTA‐high (*p* = 0.007), PTA‐mid (*p* = 0.003), and speech recognition score (*p* = 0.025) at baseline. Moreover, risk of fracture was significantly associated with worse body balance (*p* = 0.004), higher running‐speed agility (*p* = 0.012), and worse upper limb coordination (*p* = 0.044) at baseline. Both impaired hearing and balance were associated with a subsequent fracture even when consideration for multiple testing was made (Bonferroni correction). Impaired hearing, assessed by PTA, and body balance, remained significantly associated with a subsequent fracture also after adjustment for age, BMD or both combined, respectively. However, the association with speech recognition and manual coordination was no longer significant when adjusting for age and BMD. Running‐speed agility was significantly associated with a fracture when adjusted for BMD, but not when adjusted for age, or both age and BMD combined (Table 3).

**Table 3:** Hazard Ratio (HR), Unadjusted, Adjusted for Age, for BMD, and for Both Age and BMD, Calculated by Cox Regression for a Fracture During 25 Years of Follow‐Up in 80 Women With Osteoporosis

Variable	Unadjusted		Adjusted for age		Adjusted for BMD		Adjusted for age and BMD	
	HR (95% CI)	*p* Value	HR (95% CI)	*p* Value	HR (95% CI)	*P*	HR (95% CI)	*p* Value
PTA‐high (by 10), dB	1.28 (1.07– 1.53)	0.007	1.21 (1.00– 1.47)	0.049	1.27 (1.06– 1.52)	0.011	1.24 (1.01– 1.51)	0.036
PTA‐mid (by 10), dB	1.48 (1.15– 1.91)	0.003	1.39 (1.06– 1.83)	0.017	1.42 (1.08– 1.87)	0.012	1.37 (1.03– 1.82)	0.028
Speech recognition score in noise, by 1%	0.98 (0.97– 1.00)	0.025	0.99 (0.97– 1.00)	0.122	0.99 (0.97– 1.00)	0.133	0.99 (0.97– 1.01)	0.220
Body balance, by score	0.93 (0.89– 0.98)	0.004	0.94 (0.88– 1.00)	0.043	0.94 (0.89– 0.98)	0.007	0.94 (0.88– 1.00)	0.039
Running‐speed agility, by score	1.11 (1.02– 1.21)	0.012	1.09 (1.00– 1.19)	0.056	1.10 (1.01– 1.20)	0.024	1.09 (1.00– 1.19)	0.062
Upper limb coordination, by score	0.86 (0.75– 1.00)	0.044	0.89 (0.77– 1.03)	0.104	0.87 (0.76– 1.00)	0.053	0.88 (0.77– 1.02)	0.095
Manual dexterity, by score	0.94 (0.88– 1.00)	0.059	0.96 (0.89– 1.03)	0.281	0.95 (0.89– 1.01)	0.099	0.96 (0.89– 1.03)	0.291
Bone‐specific treatment during follow‐up	1.81 (1.01– 3.24)	0.047	1.90 (1.06– 3.41)	0.032	1.73 (0.96– 3.12)	0.069	1.87 (1.03– 3.38)	0.039

CI = Confidence Interval; PTA‐high = pure tone average‐high at 3, 4, and 6 kHz; PTA‐mid = pure tone average‐mid at 0.5, 1, and 2 kHz.

Fifty‐one (64%) women continued MHT the first years after the GH study was finished, but at follow‐up, 31 (61%) of these women had stopped MHT treatment. Bone‐specific treatment with bisphosphonates and/or teriparatide were given after the GH trial, mainly to those who decreased in BMD and/or fractured during follow‐up. Consequently, the HR for a fracture among women with bone‐specific treatment during the 25‐year follow‐up was nearly doubled, even after adjustment for age and BMD (Table 3).

## Discussion

The present study found that during a 25‐year follow‐up of 80 menopausal women with osteoporosis, impaired hearing, body balance, upper limb coordination, and fast running speed at baseline, were associated with a subsequent first fracture, whereas BMD was not. This is in accordance with our previous follow‐up of a random population sample of 552 women, 63 to 82 years old.^(^ ^6^ ^)^ In that study, hearing loss, but not bone‐regulating hormones, medication, or lifestyle factors, predicted incident fracture, mainly caused by accidental falls, during 17 years of follow‐up until 98 years of age.^(^ ^6^ ^)^

Postmenopausal osteoporosis is a well‐defined and strong risk factor for fractures.^(^ ^16^ ^)^ The 80 elderly women of the present study were treated with GH from 1995 through 1997. Although BMC improved up to 5‐year follow‐up in women treated with GH compared with placebo, it had returned to levels before treatment start at the 10‐year follow‐up.^(^ ^8^ ^)^ Moreover, there was no difference in the 10‐year fracture incidence between women in the GH and placebo groups.^(^ ^8^ ^)^ In this 25‐year follow‐up study of the same cohort, one or several fractures occurred in 63% of the women who participated in the GH trial, evenly distributed between the GH treatment and the placebo groups. This is somewhat higher than previously reported from Sweden,^(^ ^17^ ^)^ probably because all the women already had verified osteoporosis and/or a fracture at study start, and thereby a substantially elevated risk of fracture. The detection rate of vertebral fractures was also higher because of the repeatedly performed x‐rays in the present women. Both at baseline and at the 10‐year follow‐up presented elsewhere,^(^ ^8^ ^)^ the women of the present study had lower lean body mass than a random population sample of women of the same age who were followed in parallel during the first 10 years. A low lean body mass might, beside impaired body balance, have contributed to the fracture incidence both before and during follow‐up.

There was no association between previous GH treatment and long‐term fracture risk or death during 25 years of follow‐up. Bone mass, measured as BMD and BMC at the start of the study, did not differ between women with and without subsequent fractures during follow‐up in the present study. This contrasts with a recent Finnish study of fracture incidence in 187 healthy women aged 55 to 83 years at baseline and followed for 20 years, where BMD and BMC were significantly lower in women who fractured, whereas physical fitness did not differ between the groups.^(^ ^18^ ^)^ The relatively small sample size and long duration of follow‐up in the present study might explain the lack of association with BMD, which otherwise is an established risk factor for fracture.

In the present study, the HR for a fracture among women with bone‐specific treatment during follow‐up was nearly doubled compared with women without such treatment. However, this mirrors the likelihood that women who fractured were offered that type of treatment more often. All the women received MHT before and during the GH trial, but MHT was discontinued in almost all women during follow‐up as a consequence of the results from the Women's Health Initiative Study^(^ ^19^ ^)^ during the 1990s. This tendency of reduced use of MHT was also seen in the general population and might have contributed to the high fracture risk both in women with osteoporosis and in women in the general population.^(^ ^8^, ^20^ ^)^

The fracture panorama differs from young age to older age, with radius fractures being more common in younger ages and hip fractures more common among the elderly.^(^ ^21^ ^)^ It could be speculated that this is because of slower protective reflexes in the elderly when falling. However, the association between hearing and body balance, and its relationship with falling and fracture risk, is complex. Both sensory organs for hearing and balance are located in the inner ear; these functions are interrelated and essential for the maintenance of the postural position. Hearing impairment was already frequent at the age of 30 years in women with Turner syndrome, and was associated with impaired body balance and fine motor function.^(^ ^11^ ^)^

Although Purchase‐Helzner reported no association between hearing loss and falls or incident fractures in 6480 women aged 65 years or older in 2004,^(^ ^22^ ^)^ there is now growing evidence of a link between hearing loss and adverse health outcomes.^(^ ^23^ ^)^ The results of the present study, in a selected group of elderly women with osteoporosis, confirm the association of hearing loss and risk of fracture during 17 years of follow‐up in a random population sample of elderly Swedish women up to 98 years of age.^(^ ^6^ ^)^ Hearing loss was also significantly associated with 2‐ and 5‐year, but not 10‐year, fracture incidence, in a large retrospective study of adults over 50 years of age.^(^ ^24^ ^)^ The HR for fracture was, in the present study, significantly associated with impaired hearing, measured as PTA, also after adjustment for age and BMD. In contrast, the association to speech recognition score in noise was no longer significant after adjustments. A speculation is that PTA reflects a more peripheral hearing function, whereas the speech recognition in noise might be more dependent on central cerebral functions, and therefore more affected by increasing age.

Body balance is a complex function, and there is no golden standard measurement of overall balance. Therefore, most clinicians and researchers use a combination of tests measuring different aspects of balance. To cover the different aspects and levels of balance function several clinical tests of balance were used in this study, the simplest clinical test being Romberg's test, which is commonly used in clinical praxis worldwide. The Romberg's and sharpened Romberg's test, and the one leg stance test have all been found to have good inter‐rater and test–retest reliability.^(^ ^24^ ^)^

The BOT was originally an age‐standardized, individually administered test designed for the measurement of fine and gross motor skills of children and youths aged 4 to 21 years.^(^ ^10^ ^)^ It is intended as a discriminative and evaluative measure to characterize motor performance. In a previous study from Gothenburg, the BOT revealed a poorer fine motor function and body balance in women with Turner syndrome compared with controls with normal karyotype.^(^ ^11^ ^)^ Hence, the test discriminated well between adult patients and controls, although it is not validated in adults. Recently, the European Society for Clinical and Economic Aspects of Osteoporosis, Osteoarthritis and Musculoskeletal Diseases published a position paper recommending the use of grip strength to measure muscle strength and the use of 4‐m gait speed or the short physical performance battery test to measure physical performance in daily practice.^(^ ^26^ ^)^ Accordingly, there is an increasing awareness and concern of fall prevention measures and information in the society.

In this study, the HR for a future fracture was significantly lower among those with better body balance, also after adjustment for age and BMD. A Finnish study of middle‐aged women during 18 years of follow‐up reported similar results to ours: impaired balance (recorded as failure to stand on one foot for 10 seconds, like one part of the balance test in the present study) predicted future fracture.^(^ ^27^ ^)^ It is notable that body balance was worse in the women who died compared with the women who survived the entire follow‐up time in the present study, even if the number of fractures did not differ between them. Upper limb coordination was associated with lower HR for fracture also when adjusted for BMD, whereas adjustment for age reduced the significance. Coordination requires several complex cerebral functions that might be affected by increasing age.

Physical activity might be beneficial to prevent fall‐related fractures. A training program was recently reported to improve muscle strength, balance, and fear of falling in Norwegian women with osteoporosis and a history of vertebral fractures.^(^ ^28^ ^)^ Similarly, a randomized study of 100 Hungarian women, mean age 69 years, with menopausal osteoporosis who had at least one osteoporotic fracture, showed a significant improvement in postural balance and aerobic capacity after completing a 12‐month training program.^(^ ^29^ ^)^ Furthermore, a systematic review and meta‐analysis showed that long‐term exercise training reduced the risk of falls and tended to reduce the risk of fractures in older adults.^(^ ^30^ ^)^

A limitation of this study is the small number of participants and no update of variables tested for fracture outcome. Furthermore, the DXA device has been changed through the years, but rigorous calibrations with the phantom have been performed. Besides, all women have been compared with the same device at each occasion to minimize the methodological error. The main outcome of the present long‐term follow‐up study was fracture outcome. The strength is the thorough follow‐up with no dropouts and the long total follow‐up of 25 years by the same observer. Another strength is the objective testing with audiometry, and balance and manual coordination tests, also by a single observer. Furthermore, when and how the women fractured is well documented, including the vertebral fractures, which are often underdiagnosed and consequently underreported when assessing fracture risk with the calculator of the fracture risk assessment tool (FRAX).^(^ ^21^, ^31^ ^)^

In conclusion, hearing and body balance at baseline exceeded initial BMD in predicting incident fractures in osteoporotic women regardless of treatment during a 25‐year follow‐up. Including impaired hearing and balance function in the FRAX analysis might contribute to a more reliable calculation of a future risk of fractures, especially those by accidental falls.

## AUTHOR CONTRIBUTIONS

**Annika Dotevall:** Conceptualization; datacuration; formal analysis; writing‐original draft; Writing‐review & editing. **Emily Krantz:** Conceptualization; datacuration; writing‐original draft; writing‐review & editing. **Marie‐Louise Barrenäs:** Conceptualization;datacuration; investigation; methodology; writing‐original draft; writing‐review & editing. **Kerstin Landin‐Wilhelmsen:** Conceptualization; principal investigator; datacuration; formal analysis; funding acquisition; investigation; methodology; resources; supervision; validation.

## Conflict of Interest Disclosures

The authors have no conflicts of interest to declare.

### Peer Review

The peer review history for this article is available at https://publons.com/publon/10.1002/jbm4.10551.

## Supporting information


**Supplementary Information S1**. Bruininks–Oseretsky Test for Gross Motor Function

## Data Availability

The data that support the findings of this study are available from the authors by request.
