# Supplementary material for: Hearing and Balance Exceed Initial Bone Mineral Density in Predicting Incident Fractures: A 25‐Year Prospective Observational Study in Menopausal Women With Osteoporosis
Source: JBMR Plus. 2021 Sep 30;6(1):e10551. doi: 10.1002/jbm4.10551 (PMC8770996; doi:10.1002/jbm4.10551)
Supplement: Supplementary file 1 — Supplementary Information S1. Bruininks–Oseretsky Test for Gross Motor Function [file JBM4-6-e10551-s001.docx]

**Supplement 1: Bruininks–Oseretsky test for gross motor function**

Romberg’s test with eyes open; Romberg’s test (heel-to-toe) with eyes open

< 6 s: 0 points;
6–15 s: 1 point;
16–25 s: 2 points;
> 25 s: 3 points.

Romberg’s test with eyes closed; Romberg’s test (heel-to-toe) with eyes closed:

1–5 s: 1 point;
6–10 s: 2 points;
11–15 s: 3 points;
16–20 s: 4 points;
21–25 s: 5 points;
> 25 s: 6 points.

Standing on one leg with eyes open:

< 5 s: 0 points;
5–9 s: 1 point;
10–17 s: 2 points;
18–25 s: 3 points;
> 25 s: 4 points.

Standing on one leg with eyes closed:

< 6 s: 0 points;
6–9 s: 1 point;
10–13 s: 2 points;
14–16 s: 3 points;
17–20 s: 4 points;
21–24 s: 5 points;
25–27 s: 6 points;
> 27 s: 7 points.

Walking forward on a balance beam using a normal stride:

Crossing the first part (31 cm): 1 point;
Crossing the second part (62 cm): 2 points;
Crossing the third part (92 cm): 3 points;
Crossing the fourth part (124 cm): 4 points.
